# Alterations in bone malformation in the absence of the endosomal SNAREs Vti1a and Vti1b

**DOI:** 10.1371/journal.pone.0343070

**Published:** 2026-03-16

**Authors:** Simone Schmücker, Susanne Schöning, Christiane Wiegand, Sascha Michael Höfling, Christian Bollmann, Judith Koliwer, Gabriele Fischer von Mollard

**Affiliations:** Biochemistry III, Department of Chemistry, Bielefeld University, Bielefeld, Germany; Institut Curie, FRANCE

## Abstract

The Qb-SNAREs (soluble N-ethylmaleimide-sensitive-factor attachment receptor) Vti1a and Vti1b participate in membrane fusion in the endosomal system of mammalian cells and are partially redundant. While double deficiency of *Vti1a*^*-/-*^*Vti1b*^*-/-*^ (DKO) is perinatal lethal, double heterozygous *Vti1a*^*+/-*^*Vti1b*^*+/-*^ (DHET) mice presented no phenotypic alterations when compared to wild-type mice. To investigate the physiological role of these proteins, this study focused on the analysis of embryonic DKO versus DHET mice. The size and weight of E18.5 DKO embryos were significantly lower when compared with those of DHET littermates and wild-type embryos. Furthermore, we observed alterations in skeletal development of DKO embryos, mainly in the front limbs, ribs, clavicles and sternum. A lumbar vertebra was missing in DKO embryos and the palate was not closed in 50% of these embryos.

## Introduction

Two different mechanisms contribute to embryonic ossification in mammalian skeletons. Embryonic mesenchymal cells differentiate directly into bone-forming osteoblasts during intramembranous ossification. This process occurs mainly in the flat bones of the skull and parts of the clavicle [1–3]. In contrast, the remainder of the skeleton is formed by endochondral ossification. Here, embryonic mesenchymal cells form a cartilage anlagen, which represents the prospective bones and differentiate into chondrocytes, which undergo a specific maturation program to hypertrophic chondrocytes [1, 4, 5]. Maturation, including ossification of the cartilage anlage, is associated with the activation of various genes, chondrocyte hypertrophy, and formation of mineralized matrix [4]. Finally, both the apoptosis of hypertrophic chondrocytes and the activation of osteoclasts lead to the resorption of surrounding mineralized cartilage, allowing the formation of bone marrow invaded by blood vessels [6].

Many signaling pathways contribute to chondrocyte and bone development, such as the Wnt, Hedgehog, Fgf and transforming growth factor (TGF)‑β/bone morphogenetic protein (BMP) superfamily pathways [2, 7]. A significant amount of signaling is mediated by TGF-β or BMP receptors in endosomes after endocytosis [8]. The precise regulation of these proteins’ subcellular sorting is a prerequisite for proper functioning of these pathways. SNARE (soluble N-ethylmaleimide-sensitive-factor attachment receptor) proteins mediate the fusion of vesicles and target membranes by forming four helix-bundles, providing the driving force necessary for fusion processes [9]. The SNARE proteins contain motifs with hydrophobic amino acid residues in most of the interacting layers. The central 0‑layer consists of glutamine or arginine residues, resulting in their classification as Q- or R-SNAREs, respectively [10]. According to sequence similarities, the Q-SNAREs were further subdivided into Qa, Qb and Qc SNAREs, with one of each group and one R-SNARE being part of each SNARE complex. The Qb‑SNAREs Vti1a and Vti1b are involved in endosomal transport processes and share 30% sequence identity. Vti1a and Vti1b are expressed in all human tissues examined according to the Bgee gene expression database (https://www.bgee.org/gene/ENSG00000151532 and ENSG00000100568) as well as in mouse chondrocytes [11].

Vti1a is localized in early endosomes, the Golgi and TGN (trans-Golgi network) and forms complexes with the R‑SNARE VAMP4, the Qa‑SNAREs Syntaxin13 or Syntaxin16 and the Qc‑SNARE Syntaxin6 [12]. This complex is involved in early endosomal as well as intra-Golgi and recycling endosomal transport processes [13–16]. Vti1b, on the other hand, is present in the TGN as well as early and late endosomes [12]. Vti1b ensures the fusion with late endosomes and with lysosomes together with the Qa-SNARE Syntaxin7, the Qc-SNARE Syntaxin8 and the R-SNARE Endobrevin/VAMP8 or VAMP7, respectively [17, 18]. Mice with a knock out of either Vti1a or Vti1b display no overt phenotypes and show alterations only in some specialized cells [19, 20]. Vti1a functions in the biogenesis of secretory granules in adrenal chromaffin cells [21]. Vti1b contributes to the secretion of cytotoxic granules in T‑lymphocytes [22].

We previously described a *Vti1a*^*-/-*^*Vti1b*^*-/-*^ DKO mouse line and detected impairments in neuronal development, including the loss of neurons in ganglia as well as perinatal lethality [20, 23, 24]. Here, we analyze bone and joint formation in DKO embryos.

## Materials and methods

### Animals

*Vti1b*^*+/-*^ and *Vti1a*^*+/-*^ ES cells were generated in our laboratory as described before [19, 20]. *Vti1b*^*+/-*^ and *Vti1a*^*+/-*^ mice were housed, crossed, bred and backcrossed with C57BL/6JRccHsd mice purchased from Harlan/envigo in the mouse facility of Bielefeld University according to animal care guidelines. Mice were euthanized by CO_2_ gas inhalation and cervical dislocation in accordance with relevant guidelines and embryos were prepared on embryonic developmental day 15.5 (E15.5) or 18.5 (E18.5) after timed mating (*Vti1a*^*-/-*^ *Vti1b*^*+/-*^ or *Vti1a*^*+/-*^ *Vti1b*^*-/-*^ females with *Vti1a*^*+/-*^ *Vti1b*^*-/-*^ or *Vti1a*^*-/-*^ *Vti1b*^*+/-*^ males, respectively). All efforts were made to alleviate animal suffering. Anesthesia and/or analgesia were not required. In accordance with the German regulations for killing of vertebrates for scientific purposes §4 (3) TSchG the numbers were reported to the local administrative office. Ethical approval was not required because it is not an experiment with a living animal. The mouse strain has been deposited in the European Mouse Mutant Archive under EMMA ID 15088.

### Antibodies

**Collagen II:** (DSHB Hybridoma Product CIIC1, deposited to the DSHB by Holmdahl, R./ Rubin, K.); **Vti1a** and **Vti1b:** rabbit polyclonal serum [16]**; pan Tubulin:** rabbit polyclonal serum (Christiane Wiegand, Bielefeld University)

### Cultivation of mouse embryonic chondrocytes

For the cultivation of chondrocytes the cartilaginous parts of the rib cage of E18.5 embryos were isolated and incubated in DMEM, 200 mM L-glutamine, 10%(v/v) FCS, containing 250 µg/ml collagenase (Sigma‑Aldrich, Taufkirchen, Germany, #C9891) for 4 h at 37°C. The cells were obtained by centrifugation for 5 min at 250x g and resuspended in fresh media. The cells were incubated at 37°C and 5% CO_2_ for 3 days before use.

The quality of the chondrocyte culture was ensured via Alcian blue staining. The cells were incubated on cover slips and fixed by incubation with 100 mM glutaraldehyde in PBS (137 mM NaCl, 2.7 mM KCl, 10 mM Na_2_HPO_4_, 2 mM KH_2_PO_4_) for 20 min. Then the cells were washed with PBS three times and stained for 2 h at 20°C with 7.7 mM Alcian blue (C.I. 74240) in 3%(v/v) acetic acid. After the cells were washed once with 0.1 M HCl and twice with PBS, the cover slips were embedded in Mowiol (Sigma‑Aldrich, Taufkirchen, Germany, #81381; in 33%(v/v) glycerol).

### Alcian blue/Alizarin red staining

Embryos were isolated from the uterus and decapitated. After the removal of the skin and organs, the skeletons were incubated for 3 days in 100% ethanol, followed by incubation in 100% acetone for 24 h. Staining of the cartilage with Alcian blue and mineralized bone with Alizarin red was performed for 3 days at 37°C in staining solution (0.15 mg/ml Alcian blue (C.I. 74240), 0.05 mg/ml Alizarin red S (C.I. 58005), and 5%(v/v) acetic acid in 70% ethanol). The skeleton was washed with water for 15 min and with 178 mM KOH for 2 days and dehydrated by incubation in increasing concentrations of glycerol (2 days 20%(v/v), 2 days 50%(v/v), and 2 days 80%(v/v). Storage was performed in 100% glycerol.

### Hematoxylin/ Eosin staining

The embryos were fixed in 4% formaldehyde and embedded in paraffin. Thick sections (5–10 µM) were obtained, deparaffinized, and stained with hematoxylin and eosin.

### Immunocytochemistry

The cells were fixed with 0.04 g/ml paraformaldehyde (Carl Roth, Karlsruhe, Germany #0335) in PBS for 15 min at 20°C. After being washed two times with PBS, the cells were permeabilized with 0.02%(v/v) Triton‑X‑100 in PBS for 20 min. Again, the cells were washed two times with PBS and blocked for 30 min with 1% goat serum in PBS. The cells were incubated with a primary antibody against collagen II to detect chondrocytes in PBS/ 1% goat serum occurred for 2 h at 20°C. After two washes with PBS, cells were incubated with the secondary antibody (1:400 in PBS) for 1 h at 20°C, followed by incubation with 10 nM Hoechst 33348 for 5 min. Finally, the cells were washed three more times and embedded in Mowiol (Sigma‑Aldrich, Taufkirchen, Germany, #81381; in 33%(v/v) glycerol). Cells were analyzed with a Zeiss (Jena, Germany) LSM700 confocal microscope.

## Results

### *Vti1a*^*-/-*^*Vti1b*^*-/-*^ double deficient (DKO) embryos are characterized by the malformation of bone and joint structures

The double knockout of Vti1a and Vti1b (DKO) is perinatal lethal [20]. To ensure identical developmental age, DKO embryos should be compared with control embryos from the same mother. The probability to obtain DKO and wild-type embryos in the same litter is extremely low after mating *Vti1a*^*+/-*^*Vti1b*^*+/-*^ (DHET) mice. Therefore, we analyzed the effects of the loss of Vti1a and Vti1b on bone formation by comparing DKO (*Vti1a*^*-/-*^*Vti1b*^*-/-*^) and DHET (*Vti1a*^*+/-*^*Vti1b*^*+/-*^) embryos obtained by mating *Vti1a*^*-/-*^*Vti1b*^*+/-*^ and *Vti1a*^*+/-*^*Vti1b*^*-/-*^ mice. According to several previous studies DHET embryos show no phenotypic alterations compared to wild-type embryos [20, 23–25].

**Fig 1:**
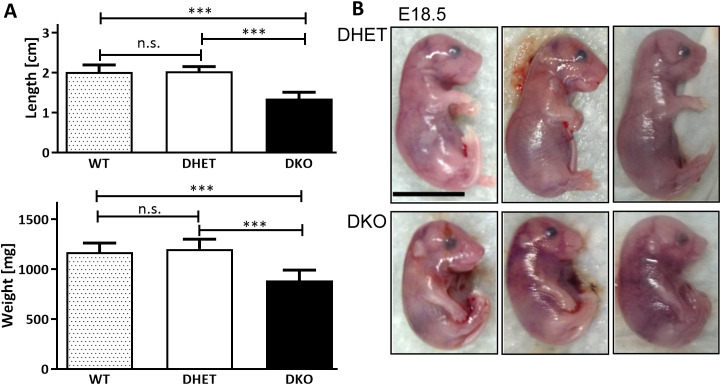
*Vti1a*^*-/-*^*Vti1b*^*-/-*^ DKO embryos were significantly smaller and lighter than *Vti1a*^*+*^^*/-*^*Vti1b*^*+*^^*/-*^ DHET littermates and were characterized by altered bending of the forelegs. **(A)** E18.5 DHET and DKO littermates and embryos derived from a wild-type mating were measured (from the crown to the base of the tail) immediately after isolation from the uterus. Compared with the size of wild-type embryos (2.01 cm) and DHET littermates (2.03 cm), the DKO were smaller (1.35 cm; n = 15) and their weight was reduced (900 mg compared to 1125 mg for wild-type and 1214 mg for DHET; n = 17) Mean + s.d.; Unpaired t‑test, two-tailed ***: p < 0.001 **(B)** E18.5 *Vti1a*^*+/-*^*Vti1b*^*+/-*^ DHET embryos held their forelegs in a typical 90° angle, whereas *Vti1a*^*-/-*^*Vti1b*^*-/-*^ DKO embryos had almost stretched forelegs. Scale bar: 1 cm.

We observed similar sizes of DKO and DHET embryos at E15.5 (see supplementary S1A Fig online, data in S2 Table online). No obvious difference was observed in their appearance at E15.5 (see supplementary S1B Fig online). In contrast, E18.5 DKO embryos were significantly smaller and lighter than both DHET littermates and wild-type embryos from a different litter (Fig 1A, data in S2 Table online). Wild-type and DHET embryos were similar in weight and size. Compared to DHET embryos, *Vti1a*^*-/-*^*Vti1b*^*+/-*^ as well as *Vti1a*^*+/-*^*Vti1b*^*-/-*^ embryos were characterized by similar sizes and weights, and these genotypes were not distinguishable in appearance (see supplementary S1C, S1D Fig online, data in S2 Table online).

**Fig 2:**
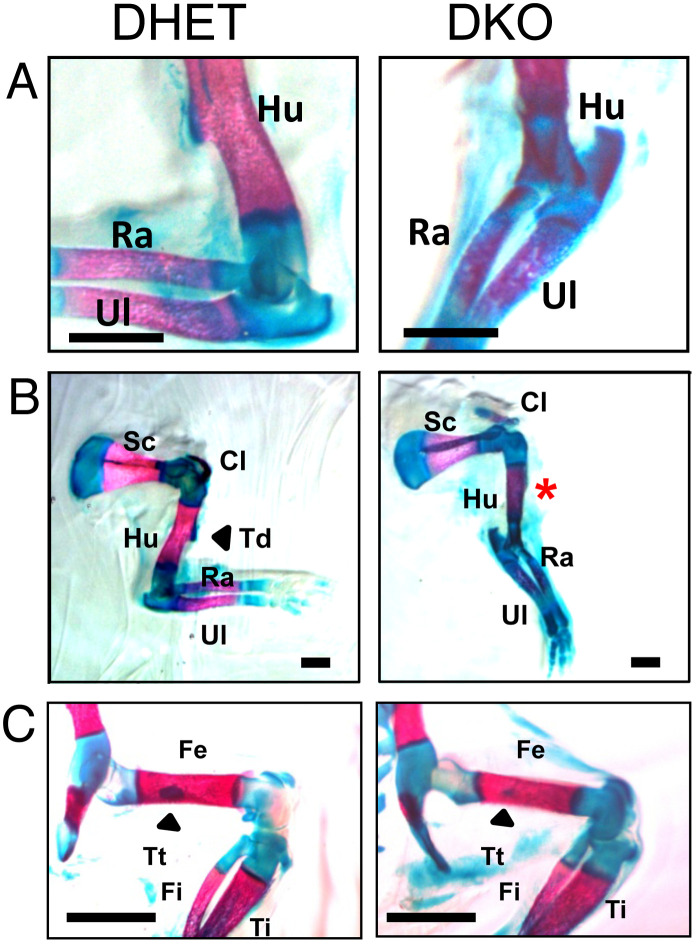
*Vti1a*^*-/-*^*Vti1b*^*-/-*^ DKO embryos presented a variety of alterations concerning bone formation in the limbs. The skeletons of E18.5 DHET and DKO embryos were stained with Alican blue (cartilage) and Alizarin red (mineralized bone). **(A)** The elbow joint socket was malformed in DKO embryos. The positions of the humerus (Hu), radius (Ra) and ulna (Ul) were altered. At E18.5, 72% of DKO embryos, but none of DHET embryos showed this abnormality. **(B)** A well-developed deltoid tuberosity (Td) was missing on the humerus of DKO embryos. Sc: scapula. **(C)** The third trochanter (Tt) was less pronounced on the femur (Fe) in the hind leg of DKO embryos. Fi: fibula, Ti: tibia. Scale bar: 1 mm. DHET: n = 25, DKO: n = 28.

**Fig 3:**
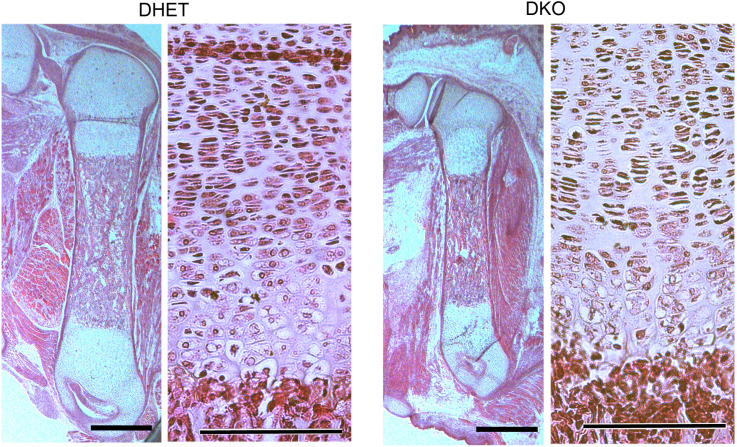
Chondrocytes in the femurs of *Vti1a*^*-/-*^*Vti1b*^*-/-*^ DKO and DHET embryos developed similarly. Sections were prepared from E18.5 DHET and DKO embryos and stained with hematoxylin and eosin. The femur (left panel) and chondrocytes in the growth plate (right panel) looked similarly in DHET and DKO embryos. Scale bars: 500 µm (left panel) and 150 µm (right panel).

At E18.5 severe differences were observed concerning the bending of the forelegs of the embryos (Fig 1B). Whereas DHET embryos held their forelegs at a 90° angle at the elbow joint, DKO embryos had nearly stretched forelegs. Interestingly, this observation corresponds to alterations of the elbow bone and joint structures (Fig 2A). In 72% of DKO embryos we observed a malformation of the elbow joint socket, which was associated with impaired anchoring of the humerus. DKO embryos were furthermore characterized by malpositioning of the ulna and radius. To compare these observations with those of earlier stages of development, we also analyzed E15.5 DKO versus DHET embryos. Interestingly, E15.5 DKO embryos presented only minor alterations in foreleg bending with low penetrance (see supplementary S1B Fig online), and indeed, only 20% exhibited alterations in the elbow joint. The deltoid tuberosity is a bone ridge on the humerus that serves as the attachment for the deltoid muscle. Its development by endochondral ossification is initiated by the tendon and it grows due to muscle contractions [26]. The deltoid tuberosity was missing in 32% and was reduced in 57% of DKO embryos (Fig 2B). The third trochanter, a muscle attachment area on the femur, was reduced in 43% of the DKO embryos (Fig 2C). Next, we studied the femurs in more detail in tissue sections. The overall appearance of the femur and the chondrocytes at the growth plate looked similar in E18.5 DHET and DKO embryos (Fig 3).

**Fig 4:**
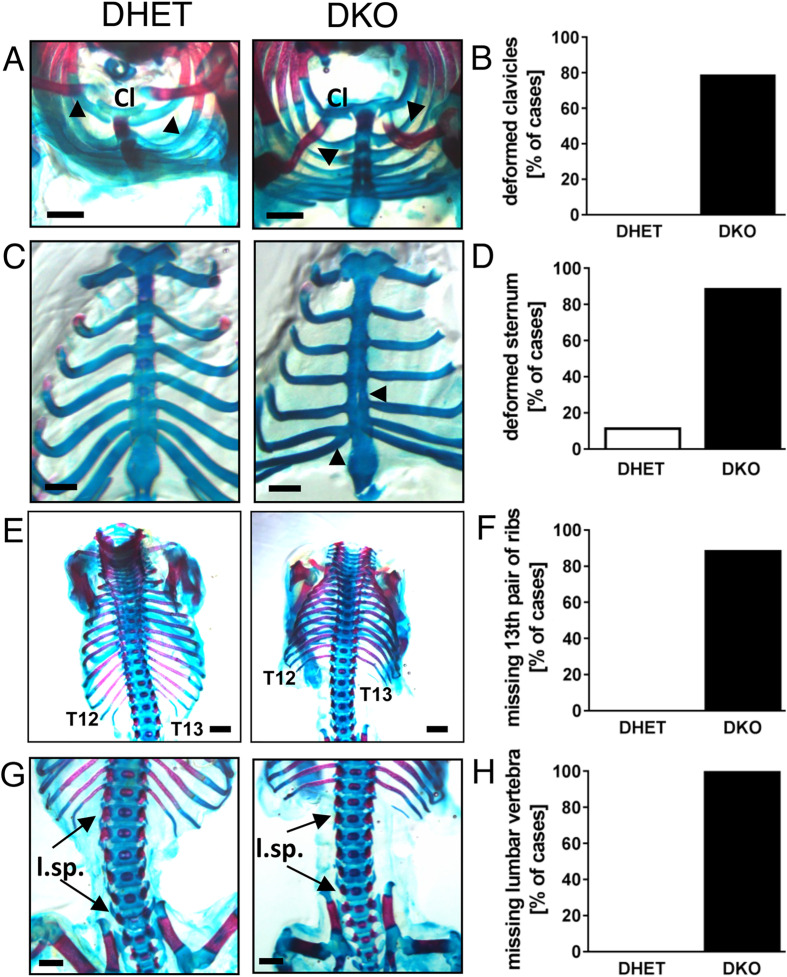
*Vti1a*^*-/-*^*Vti1b*^*-/-*^ DKO embryos presented a variety of alterations concerning bone formation in clavicles, sternum, ribs and vertebrae. The skeletons of E18.5 DHET and DKO embryos were stained with Alican blue (cartilage) and Alizarin red (mineralized bone). **(A)** DKO embryos presented deformed clavicles (Cl), compared to straight ones observed in DHET embryos. **(B)** This observation was made in 79% of DKO embryos at E18.5. **(C)** At E18.5, the calcification of the sternum was further progressed in DHET embryos compared with DKO embryos. **(D)** 89% of DKO, but only 12% of DHET embryos presented malformation of the sternum. **(E)** The 13^th^ pair of ribs was symmetric and partly calcified in DHET embryos at E18.5. In contrast, in DKO embryos either missing structures or only cartilage anlages of two complete ribs have been found at this position. **(F)** 89% of DKO embryos, but none of the investigated DHET embryos presented one or two missing ribs at the T13 vertebra. **(G, H)** In contrast to the 6 vertebrae of the lumbar spine (l.sp.) in DHET embryos, only 5 lumbar vertebrae were detected in all DKO embryos. Scale bar: 1 mm. DHET: n = 25, DKO: n = 28.

A detailed investigation revealed that *Vti1a*^*-/-*^*Vti1b*^*-/-*^ DKO embryos show a variety of additional abnormalities related to bone formation. Alterations were detected in the thorax of DKO embryos. In contrast to straight clavicles in DHET embryos, deformed clavicles were observed in 80% of DKO embryos at E18.5 (Fig 4A, 4B). However, corresponding alterations were detected in only 20% of DKO embryos at E15.5. Furthermore, malformation of the sternum, associated with impaired calcification, was detected in 89% of DKO embryos, but in only 12% of DHET embryos (Fig 4C, 4D). Calcification was absent at E15.5 DHET and DKO embryos. Moreover, 89% of the E18.5 DKO embryos missed one or both ribs at the T13 vertebra (Fig 4E, 4F). In some animals, we found rudimentary structures instead. In all the analyzed DKO embryos, which had two complete ribs at the 13^th^ vertebra, calcification was missing completely and only cartilage was found at this position. In general, this calcification was completed in DHET embryos. In only 60% of the cases, the form and symmetry of the 13^th^ pair of ribs of E15.5 DKO embryos were altered (see supplementary S2 Fig online). Interestingly, the remaining DKO embryos lacked initial calcification at this position, whereas calcification generally started in DHET embryos. This led to the assumption that specific regulatory processes involved in the calcification of cartilage anlages were impaired, leading to malformation of the rib pair after E15.5. E15.5 as well as E18.5 DKO embryos lacked one of the lumbar vertebrae, which was present in all DHET embryos (Fig 4G, 4H). This observation is consistent with the development of the lumbar spine from the sclerotome of somites starting at E10 [27]. Interestingly, we also observed a missing lumbar vertebra also in 12% of the *Vti1a*^*-/-*^*Vti1b*^*+/-*^ embryos but not in the *Vti1a*^*+/-*^*Vti1b*^*-/-*^ embryos (see supplementary S3 Fig online). A deformed sternum was observed with low penetrance in *Vti1a*^*-/-*^*Vti1b*^*+/-*^ and *Vti1a*^*+/-*^*Vti1b*^*-/-*^ embryos.

**Fig 5:**
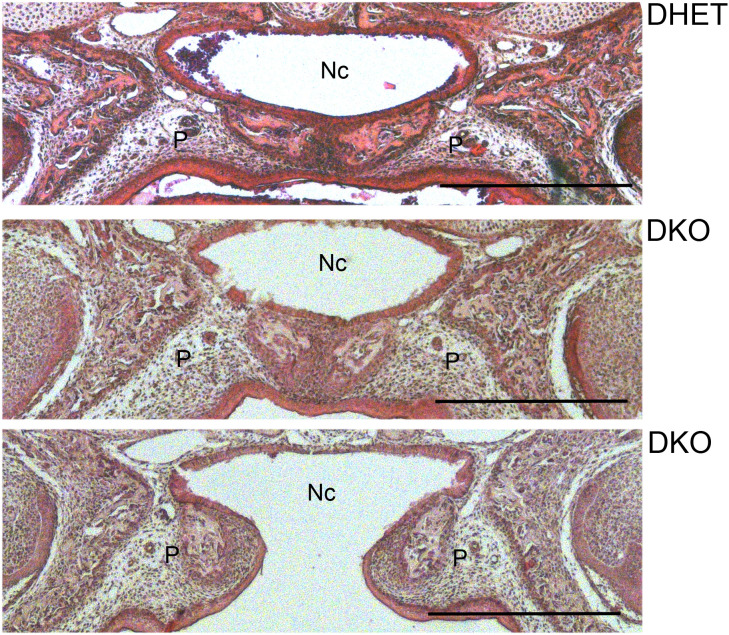
The palate was not closed in 50% of *Vti1a*^*-/-*^*Vti1b*^*-/-*^ DKO embryos. Coronal sections of the heads were prepared from E18.5 DHET and DKO embryos and stained with hematoxylin and eosin. The palate (P) was not closed in 50% of DKO embryos but was closed in all DHET embryos. Nc: nasal cavity, P: palate. Scale bar: 500 µm. DHET: n = 6, DKO: n = 10.

Analysis of the craniofacial structures revealed that the palate was not closed in 50% of the DKO embryos but was closed in all DHET littermates (Fig 5). The palate was also affected in approximately 10% of the in *Vti1a*^*-/-*^*Vti1b*^*+/-*^ and *Vti1a*^*+/-*^*Vti1b*^*-/-*^ embryos (see supplementary S3 Fig online).

To analyze protein levels, cartilage was prepared from the cartilaginous parts of the rib cage of DKO and DHET E18.5 embryos and used to cultivate chondrocytes. We verified the quality of the chondrocyte cultures in comparison to fibroblasts by their production of acidic proteoglycans stained with Alcian blue (see supplementary S4A Fig online) and the chondrocyte marker collagen II via the use of specific antibodies in immunocytochemistry (see supplementary S4B Fig online). Vti1a and Vti1b were detected in western blots with protein samples from DHET chondrocytes demonstrating that these SNAREs are present in chondrocytes as expected (see supplementary S4C Fig). Vti1a and Vti1b were not observed in DKO samples confirming the knockout (see supplementary S4D Fig online).

## Discussion

*Vti1a*^*-/-*^*Vti1b*^*-/-*^ DKO embryos presented phenotypic abnormalities related to bone formation. The majority of these alterations involved parts of the skeleton, which are formed by endochondral ossification, such as the ribs, sternum or front limbs. We compared the phenotype of *Vti1a*^*-/-*^*Vti1b*^*-/-*^ DKO embryos with the phenotype of mice lacking proteins in signaling pathways associated with bone formation and detected interesting parallels with those of *Tgf‑b2*^*-/-*^ KO, *Tgf‑b2*^*-/-*^*Tgf‑b3*^*-/-*^ DKO, *TGF‑b2*^*+/-*^*TGF‑b3*^*-/-*^ KO and *Bmp‑7*^*-/-*^ KO mice [28–31], while other phenotypic observations were different between the genotypes (S1 Table). Most of the mice were viable through embryogenesis and died shortly before or after birth. *Vti1a*^*-/-*^*Vti1b*^*-/-*^ DKO embryos shared the phenotype of reduced body size, malposition of the limbs, alterations in rib pairing, sternum malformations, deformation of the clavicles, and cleft palate with *Tgf‑b2*^*-/-*^ KO mice. *Tgf‑b2*^*-/-*^*Tgf‑b3*^*-/-*^ DKO embryos die at E15.5 with alterations in the lower rib pairs and malformation of the sternum. *Vti1a*^*-/-*^*Vti1b*^*-/-*^ DKO and *Tgf‑b2*^*+/-*^*Tgf‑b3*^*-/-*^ embryos both display cleft palate. BMP-7 is associated with embryonic bone development and induces osteoblastic differentiation [32, 33]. *BMP-7*^*-/-*^ KO mice display a missing lumbar vertebra and a malformation of the 13^th^ pair of ribs similar to that of *Vti1a*^*-/-*^*Vti1b*^*-/-*^ DKO embryos [30, 31].

The absence of the endosomal SNAREs Vti1a and Vti1b could alter the transport kinetics between the plasma membrane and endosomes. TGF‑β and BMPs require a combination of type I and II receptors for effective binding, which can be modulated by coreceptors [34]. These receptors are constitutively endocytosed via clathrin coated vesicles and recycled after passage through endosomes [8, 35]. They can be endocytosed via cholesterol/caveolae-rich domains and targeted for degradation [34]. Therefore, these signaling pathways could be more sensitive to the proper subcellular distribution of their receptors. In addition, clathrin mediated endocytosis enhances TGF-ß induced Smad activation [8]. Different scaffold proteins are found on endosomes, which bind to receptors and signaling proteins allowing for crosstalk between different signaling pathways [36].

Finally, the secretion of signaling peptides and proteins during embryonic development is a crucial part of the regulation of bone formation. It has been described previously that Vti1a, but not Vti1b, contributes to the biogenesis of secretory granules in adrenal chromaffin cells [21], suggesting that the loss of Vti1a could reduce the secretion of relevant signaling molecules. This hypothesis is supported by a higher penetrance of phenotypic alterations in *Vti1a*^*-/-*^*Vti1b*^*+/-*^ embryos compared to *Vti1a*^*+/-*^*Vti1b*^*-/-*^ embryos. However, secretion can be responsible for only a small portion of the alterations, because the penetrance in DKO embryos is so much higher than that in both of these genotypes.

Egfr^-/-^ KO mice (Epidermal growth factor receptor) share the cleft palate with Vti1a^-/-^Vti1b^-/-^ embryos [37]. Impaired PI3K-mediated PDGFRα signaling (Platelet-derived growth factor receptor) is associated with rib malformation and cleft palate [38, 39], indicating that impairments in several central signaling pathways result in skeletal abnormalities. TGF-ß and PDGF signaling influence each other during vertebral closure [40].

*TGF-b2*^*-/-*^ KO embryos are also lack the deltoid tuberosity and the third trochanter [28]. *Vti1a*^*-/-*^*Vti1b*^*-/-*^ DKO and *TGF-b2*^*-/-*^ KO embryos display no voluntary movement and an extended posture of the forelimb [41], which is typical for embryos with defects in muscle contraction [42]. Therefore, lack or reduction of the deltoid tuberosity and the third trochanter could be due to the initiation of these structures by endochondral ossification or growth, which requires muscle contraction [26].

Taken together, our data indicate that the double knockout of Vti1a and Vti1b resulted in the malformation of bones and joints in the embryonic mouse skeleton. *Vti1a*^*-/-*^*Vti1b*^*+/-*^ and *Vti1a*^*+/-*^*Vti1b*^*-/-*^ embryos were associated with mild skeletal alterations with very low penetrance and are viable and fertile. Both proteins can replace each other in most functions. We speculate that signaling pathways in bone formation might be affected by the loss of the SNAREs Vti1a and Vti1b.

## Supporting information

S1 Fig *Vti1a*^*-/-*^*Vti1b*^*-/-*^ DKO at E15.5 and *Vti1a*^*-/-*^*Vti1b*^*+/-*^ or *Vti1a*^*+/-*^*Vti1b*^*-/-*^ at E18.5 are devoid of alterations in size or obvious abnormalities of the body structure. (A) E15.5 DHET and DKO were measured immediately after isolation from the uterus. The size of DKO was not altered compared to DHET. (N = 15) (B) No obvious difference was observed between E15.5 DHET and DKO mice. Scale bar: 5 mm (C) *Vti1a*^*-/-*^*Vti1b*^*+/-*^ and *Vti1a*^*+/-*^*Vti1b*^*-/-*^ are not associated with differences in the size (N = 15) or weight (*Vti1a*^*-/-*^*Vti1b*^*+/-*^: N = 8, *Vti1a*^*+/-*^*Vti1b*^*-/-*^: N = 5) of E18.5 embryos compared to DHET. (D) *Vti1a*^*-/-*^*Vti1b*^*+/-*^ and *Vti1a*^*+/-*^*Vti1b*^*-/-*^ mice show only minor obvious phenotypic alterations in E18.5 embryos. Scale bar: 1 cm. (PDF)

S2 Fig Absent or less calcified T13 ribs in E15.5 *Vti1a*^*-/-*^*Vti1b*^*-/-*^ DKO embryos. Skeletons of E15.5 DHET and DKO embryos stained with Alican blue (cartilage) and Alizarin red (mineralized bone) viewed from the dorsal side. (A) Calcification started in the ribs at the T13 vertebra of DHET embryos. (B) DKO embryos with ribs at T13 lacked initial calcification at this position. (C) Ribs at the T13 vertebra were lacking, altered in form or symmetry in 60% of E15.5 embryos. Scale bar: 1 mm. DHET: N = 16, DKO N = 10. (PDF)

S3 Fig Only minor alterations in specific bone structures can be observed with low penetrance in *Vti1a*^*-/-*^*Vti1b*^*+/-*^ and *Vti1a*^*+/-*^*Vti1b*^*-/-*^ embryos. With reduced manifestation, similar phenotypic observations of *Vti1a*^*-/-*^*Vti1b*^*-/-*^ DKO could be found in *Vti1a*^*-/-*^*Vti1b*^*+/-*^ and *Vti1a*^*+/-*^*Vti1b*^*-/-*^ embryos with very low penetrance, concerning elbow joint (A), palate cleft (B), clavicles (C), sternum (D), and lumbar spine (E). Scale bar: 1 mm. (B, D, E) *Vti1a*^*-/-*^*Vti1b*^*+/-*^: N = 28, *Vti1a*^*+/-*^*Vti1b*^*-/-*^: N = 21 (C) *Vti1a*^*-/-*^*Vti1b*^*+/-*^: N = 25, *Vti1a*^*+/-*^*Vti1b*^*-/-*^: N = 17. (PDF)

S4 Fig Vti1a and Vti1b are present in DHET but not DKO chondrocytes. Chondrocytes were cultivated from the cartilaginous parts of the rib cage of DHET and DKO E18.5 embryos. (A) The quality of the chondrocyte culture was verified via Alcian blue staining. Chondrocytes synthesize acidic proteoglycans, which produce a blue signal with Alcian blue. (B) Collagen II secreted by chondrocytes was stained with specific antibodies. In comparison, mouse embryonic fibroblasts were negative for Alcian blue and collagen II staining. Scale bar: as indicated. Proteins were separated by SDS-PAGE and analyzed by Western blotting for Vti1a (C) or Vti1b (D). Antibodies directed against tubulin were used as loading control. (PDF)

S1 Table Comparison of phenotypic alterations due to loss of Vti1a and Vti1b or known important regulatory components concerning bone formation. (PDF)

S2 Table Data file with size and weight of embryos. (XLSX)

S1_raw_images Raw images of western blots shown in S4 Fig. (PDF)
