# Supplementary material for: Alterations in bone malformation in the absence of the endosomal SNAREs Vti1a and Vti1b
Source: PLoS One. 2026 Mar 16;21(3):e0343070. doi: 10.1371/journal.pone.0343070 (PMC12991266; doi:10.1371/journal.pone.0343070)
Supplement: S1 Fig — (PDF) [file pone.0343070.s001.pdf]

Fig. S1

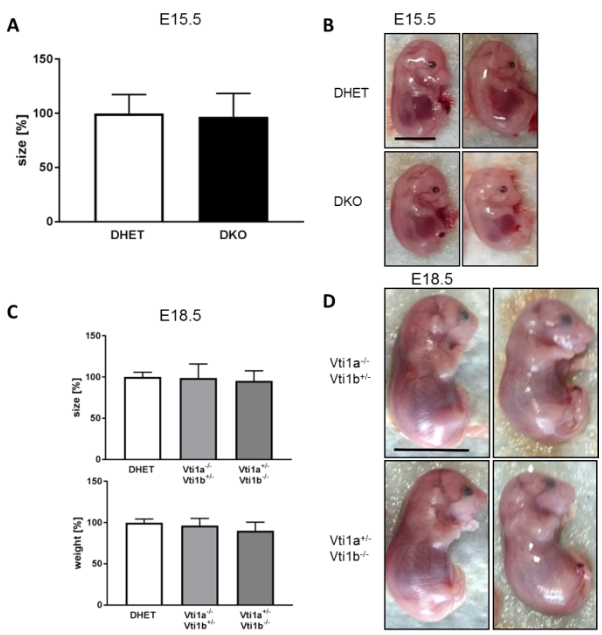

Fig. S1: *Vti1a$^{-/-}$Vti1b$^{-/-}$* DKO at E15.5 and *Vti1a$^{-/-}$Vti1b$^{+/-}$* or *Vti1a$^{+/-}$Vti1b$^{-/-}$* at E18.5 are devoid of alterations in size or obvious abnormalities of the body structure.

(A) E15.5 DHET and DKO were measured immediately after isolation from the uterus. The size of DKO was not altered compared to DHET. (N=15) **(B)** No obvious difference was observed between E15.5 DHET and DKO mice. Scale bar: 5 mm **(C)** *Vti1a$^{-/-}$Vti1b$^{+/-}$* and *Vti1a$^{+/-}$Vti1b$^{-/-}$* are not associated with differences in the size (N=15) or weight (*Vti1a$^{-/-}$Vti1b$^{+/-}$*: N=8, *Vti1a$^{+/-}$Vti1b$^{-/-}$*: N=5) of E18.5 embryos compared to DHET. **(D)** *Vti1a$^{-/-}$Vti1b$^{+/-}$* and *Vti1a$^{+/-}$Vti1b$^{-/-}$* mice show only minor obvious phenotypic alterations in E18.5 embryos. Scale bar: 1 cm
