# Supplementary material for: Alterations in bone malformation in the absence of the endosomal SNAREs Vti1a and Vti1b
Source: PLoS One. 2026 Mar 16;21(3):e0343070. doi: 10.1371/journal.pone.0343070 (PMC12991266; doi:10.1371/journal.pone.0343070)
Supplement: S2 Fig — (PDF) [file pone.0343070.s002.pdf]

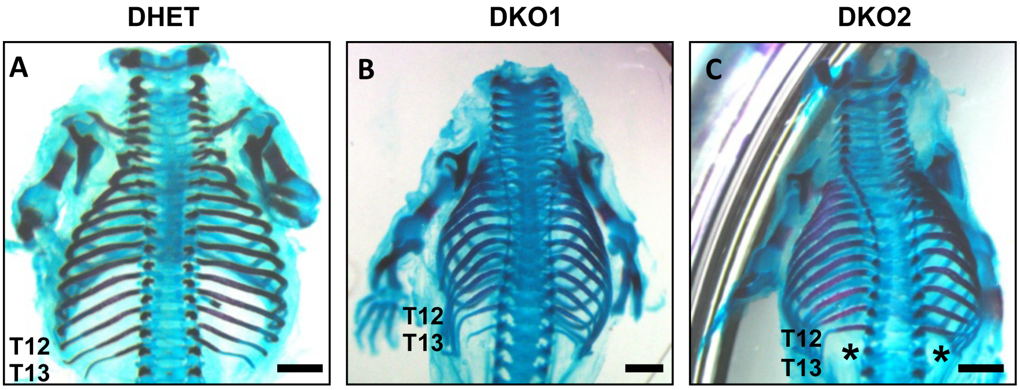

Fig. S2: Absent or less calcified T13 ribs in E15.5 *Vti1a$^{-/-}$Vti1b$^{-/-}$* DKO embryos.

Skeletons of E15.5 DHET and DKO embryos stained with Alican blue (cartilage) and Alizarin red (mineralized bone) viewed from the dorsal side. **(A)** Calcification started in the ribs at the T13 vertebra of DHET embryos. **(B)** DKO embryos with ribs at T13 lacked initial calcification at this position. **(C)** Ribs at the T13 vertebra were lacking, altered in form or symmetry in 60% of E15.5 embryos. Scale bar: 1 mm. DHET: N=16, DKO N=10
