# Supplementary material for: Alterations in bone malformation in the absence of the endosomal SNAREs Vti1a and Vti1b
Source: PLoS One. 2026 Mar 16;21(3):e0343070. doi: 10.1371/journal.pone.0343070 (PMC12991266; doi:10.1371/journal.pone.0343070)
Supplement: S3 Fig — (PDF) [file pone.0343070.s003.pdf]

Fig. S3

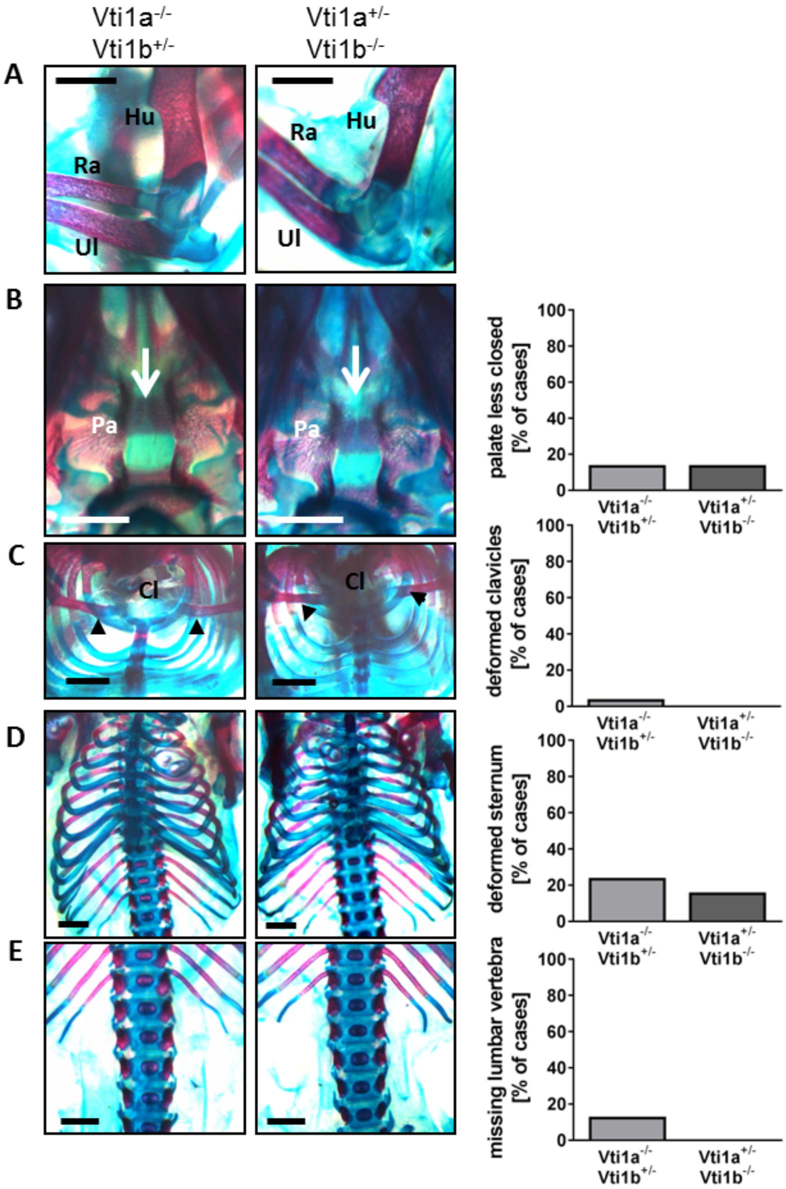

Fig. S3: Only minor alterations in specific bone structures can be observed with low penetrance in *Vti1a$^{-/-}$Vti1b$^{+/-}$* and *Vti1a$^{+/-}$Vti1b$^{-/-}$* embryos.

With reduced manifestation, similar phenotypic observations of *Vti1a$^{-/-}$Vti1b$^{-/-}$* DKO could be found in *Vti1a$^{-/-}$Vti1b$^{+/-}$* and *Vti1a$^{+/-}$Vti1b$^{-/-}$* embryos with very low penetrance, concerning elbow joint **(A)**, palate cleft **(B)**, clavicles **(C)**, sternum **(D),** and lumbar spine **(E)**. Scale bar: 1 mm. **(B, D, E)** *Vti1a$^{-/-}$Vti1b$^{+/-}$*: N=28, *Vti1a$^{+/-}$Vti1b$^{-/-}$*: N=21 **(C)** *Vti1a$^{-/-}$Vti1b$^{+/-}$*: N=25, *Vti1a$^{+/-}$Vti1b$^{-/-}$*: N=17
