# Supplementary material for: Alterations in bone malformation in the absence of the endosomal SNAREs Vti1a and Vti1b
Source: PLoS One. 2026 Mar 16;21(3):e0343070. doi: 10.1371/journal.pone.0343070 (PMC12991266; doi:10.1371/journal.pone.0343070)
Supplement: S4 Fig — (PDF) [file pone.0343070.s004.pdf]

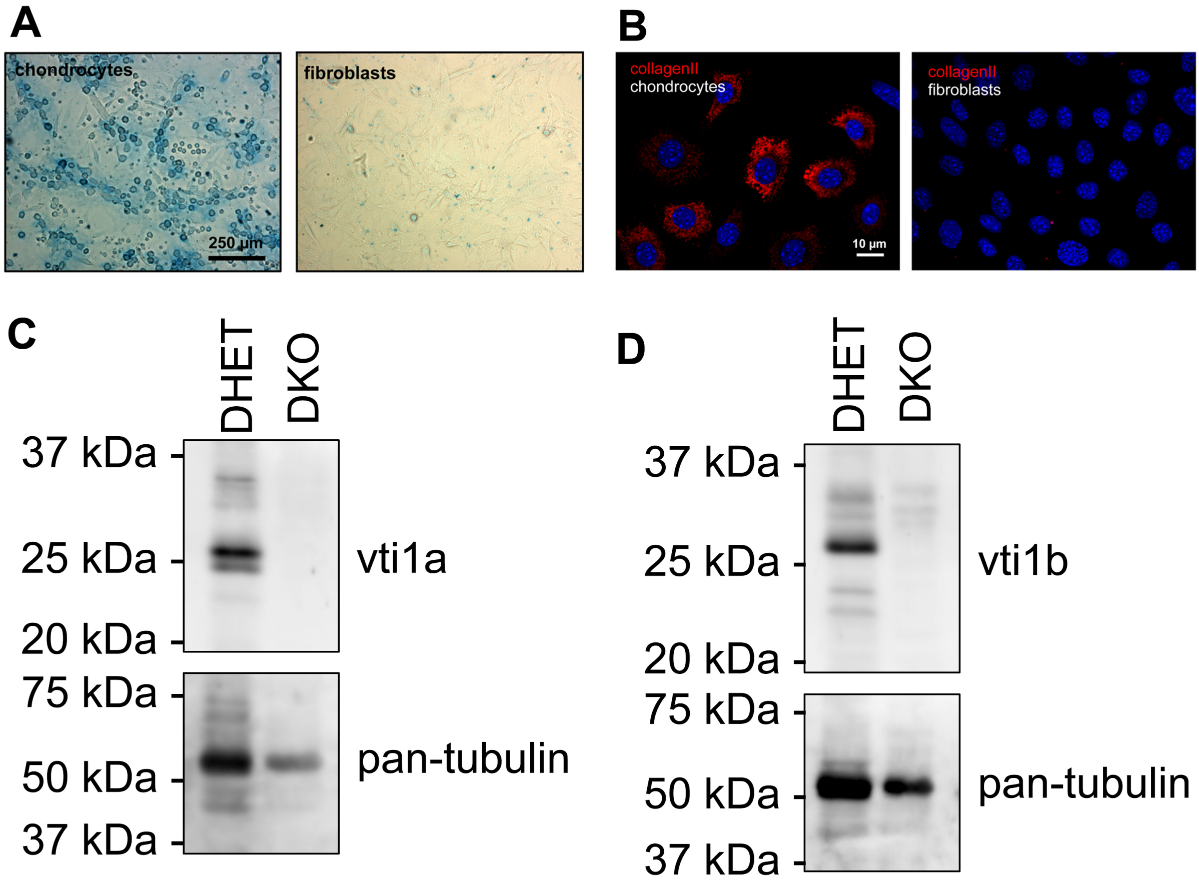

Fig. S4: Vti1a and Vti1b are present in DHET but not DKO chondrocytes.

Chondrocytes were cultivated from the cartilaginous parts of the rib cage of DHET and DKO E18.5 embryos. **(A)** The quality of the chondrocyte culture was verified via Alcian blue staining. Chondrocytes synthesize acidic proteoglycans, which produce a blue signal with Alcian blue. **(B)** Collagen II secreted by chondrocytes was stained with specific antibodies. In comparison, mouse embryonic fibroblasts were negative for Alcian blue and collagen II staining. Scale bar: as indicated. Proteins were separated by SDS-PAGE and analyzed by Western blotting for Vti1a **(C)** or Vti1b **(D)**. Antibodies directed against tubulin were used as loading control.
