# Supplementary material for: Alterations in bone malformation in the absence of the endosomal SNAREs Vti1a and Vti1b
Source: PLoS One. 2026 Mar 16;21(3):e0343070. doi: 10.1371/journal.pone.0343070 (PMC12991266; doi:10.1371/journal.pone.0343070)
Supplement: S1 Table — (PDF) [file pone.0343070.s005.pdf]

**Table S1: Comparison of phenotypic alterations due to loss of Vti1a and Vti1b or known important regulatory components concerning bone formation**

**++**: observed in more than 50% of cases, **+**: observed, penetrance not indicated, **(+)**: observed in 10-50% of cases, **--**: observed in less than 10%, **n. i.**: not investigated

| | $Vti1a^{-/-}$ $Vti1b^{-/-}$ | $Tgf\text{-}ß2^{-/-}$ (28, 39) | $Tgf\text{-}ß2^{-/-}$ $Tgf\text{-}ß3^{-/-}$ (29) | $Tgf\text{-}ß2^{+/-}$ $Tgf\text{-}ß3^{-/-}$ (29) | $Bmp\text{-}7^{-/-}$ (30, 31) |
|---|---|---|---|---|---|
| Lethality | perinatal | perinatal | prenatal | perinatal | perinatal |
| Lower weights at | ++ (E18.5) | ++ (E18.5) | n. i. | + (E18.5) | + (P0) |
| Malposition of the limbs | ++ | ++ | n. i. | -- | -- |
| Missing one of the lumbar vertebrae | + + | n. i. | n. i. | -- | + |
| Malformations of rib cage | ++ | ++ | + | -- | ++ |
| Reduced 13th pair of ribs | ++ | -- | -- | -- | + |
| Sternum malformations | ++ | (+) | + | -- | + |
| Delayed calcification of sternum | ++ | n. i. | n. i. | -- | n. i. |
| Deformation of clavicles | ++ | ++ | n. i. | -- | -- |
| Cleft palate | (+) | (+) | + | + | -- |
