# Supplementary figures and images for: Alterations in bone malformation in the absence of the endosomal SNAREs Vti1a and Vti1b
Source: PLoS One. 2026 Mar 16;21(3):e0343070. doi: 10.1371/journal.pone.0343070 (PMC12991266; doi:10.1371/journal.pone.0343070)

X X

**Fig S4C**

**Vti1a**

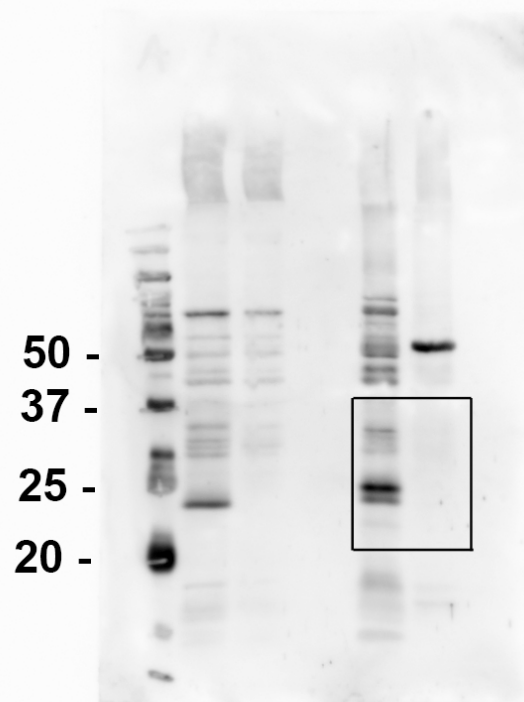

**Fig S4 C**

**pan-tubulin**

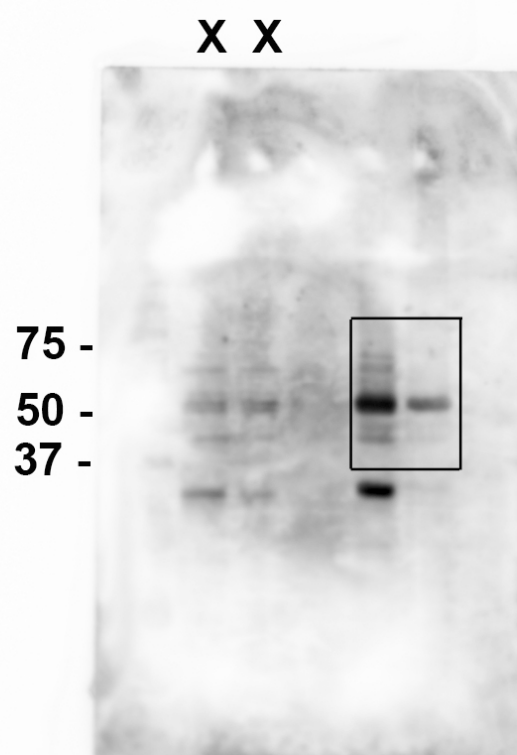

**Fig. S4 D**

**Vti1b**

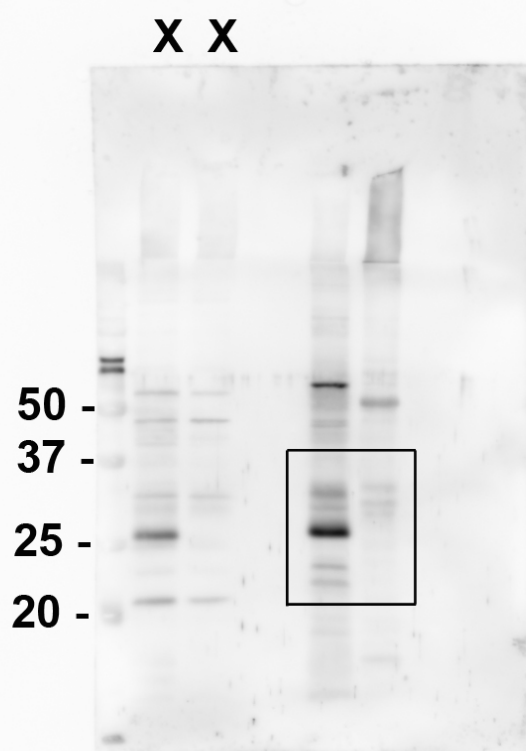

**Fig. S4 D**

**pan-tubulin**

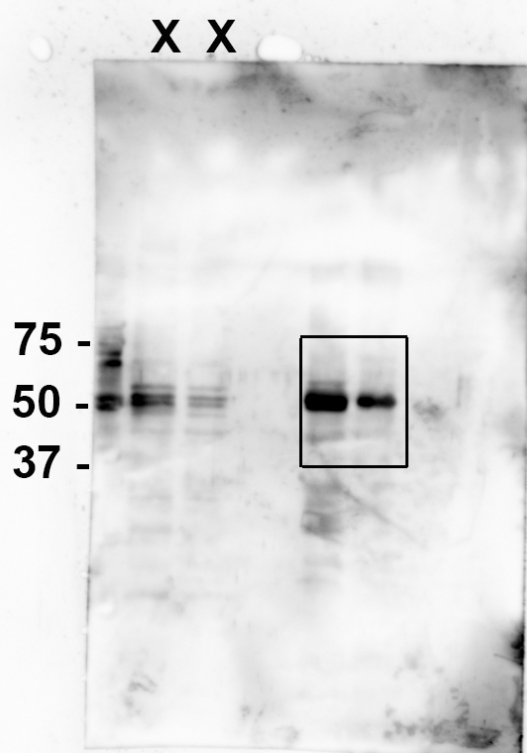

Supplement: S1_raw_images — (PDF) [file pone.0343070.s007.pdf]
